# Difference in medical service use among Korean gastric cancer survivors according to regional healthcare vulnerabilities: a cohort study

**DOI:** 10.1007/s00520-022-07346-2

**Published:** 2022-09-03

**Authors:** Sung Hoon Jeong, Jae Hong Joo, Minah Park, Choa Yun, Soo Hyun Kang, Eun-Cheol Park, Yoon Dae Han, Sung-In Jang

**Affiliations:** 1 Department of Public Health, Graduate School, Yonsei University, Seoul, Republic of Korea; 2 Institute of Health Services Research, Yonsei University, Seoul, Republic of Korea; 3 Department of Biostatistics & Computing, College of Medicine, Yonsei University, Seoul, Republic of Korea; 4 Department of Preventive Medicine, Yonsei University College of Medicine, Seoul, Republic of Korea; 5 Department of Surgery, Severance Hospital, Yonsei University College of Medicine, Seoul, Republic of Korea

**Keywords:** Gastric cancer, Medical service use, Healthcare vulnerability, Regional disparities, Korea

## Abstract

**Purpose:**

This study investigated the relationship between medical service use and healthcare vulnerability, pre- and post-gastric cancer diagnosis. Differences between healthcare-vulnerable and healthcare-nonvulnerable regions identified inequities that require intervention.

**Methods:**

This cohort study was done using the National Health Insurance claims data of patients diagnosed with gastric cancer between 2004 and 2013. The Position Value for Relative Comparison Index was used to determine whether the patients lived in a healthcare-vulnerable region. Medical service use was classified into annual outpatient treatment, hospitalization days, and emergency treatment. We used a generalized linear model to which the Poisson distribution was applied and compared regional differences in medical service use.

**Results:**

A total of 1797 gastric cancer patients who had survived 5 years post-diagnosis were included in the study, of which 14.2% lived in healthcare-vulnerable regions. The patients in vulnerable regions surviving 5–7 years post-diagnosis had a higher number of outpatient visits than those in nonvulnerable regions. Furthermore, hospitalization days were lesser for patients in vulnerable regions who survived 6 years post-diagnosis than those in nonvulnerable regions; however, this number increased in the seventh year.

**Conclusions:**

Our results suggest that gastric cancer survivors living in healthcare-vulnerable regions have a higher probability of increased medical service use 5 years post-diagnosis compared with patients in nonvulnerable regions, which may significantly increase healthcare disparities over time. Therefore, in the future, additional research is needed to elucidate the causes of the disparities in healthcare use and the results of the differences in health outcomes.

**Supplementary Information:**

The online version contains supplementary material available at 10.1007/s00520-022-07346-2.

## Introduction

Gastric cancer is a major health problem and a leading cause of cancer-related mortality, making it a significant public health issue [1, 2]. Globally, gastric cancer is the fifth most common disease, accounting for 5.7% of new cancer cases, and according to GLOBOCAN 2018 data, it is the third most fatal cancer estimated to have killed 782,685 people worldwide [3]. Although the incidence of gastric cancer has been steadily decreasing in recent years, it continues to remain high in Central Asia, Latin America, and East Asian countries, including South Korea, along with the associated mortality rate [4]. In particular, the incidence of gastric cancer in South Korea has been gradually decreasing over the past decade; nevertheless, malignant tumors related to gastric cancer rank first in cancer-related incidence and fourth in mortality [5, 6]. Fortunately, as Korea’s national cancer screening program has been actively conducted in recent years, most patients with gastric cancer could be diagnosed at an early stage [7]. Consequently, the 5-year relative survival rate of individuals with gastric cancer in Korea was 76.0% during 2012–2016, an increase of 33.2%, compared to what it was 20 years ago [8].

Korea has achieved these results by focusing on prevention and treatment through a multifaceted national cancer management policy. However, cancer survivors experience various physical and psychosocial problems, as well as adverse symptoms caused by cancer treatment [9]. During the course of cancer treatment, patients usually receive various high-intensity treatments, including surgery, chemotherapy, radiation therapy, and hormone therapy—leading to symptoms such as nausea, vomiting, and pain for a considerable period of time immediately after the treatment phase [10]. Even if the cancer is cured after such acute treatment, long-term sequelae or body changes are common [10]. Therefore, cancer patients require social and medical support from a variety of healthcare providers throughout the disease trajectory [11].

Such issues are becoming more important from the viewpoint of equity of medical service use between urban and rural areas [12]. The reason why equity between regions is important in the use of medical services is that the latter can be directly related to health outcomes [13]. Despite the need for more medical assistance in rural areas of Korea, the required resources and medical expenditures are more concentrated in large cities [14]. Therefore, gastric cancer patients living in rural areas are reported to be at higher risk of morbidity, hospitalization, and death compared with those living in urban areas [15]. Moreover, rural residents are likely to have lower incomes and greater difficulty in accessing medical and necessary support services owing to the limited number of medical facilities [15, 16].

Although interest in cancer survivors has recently been increasing, studies on cancer survivors mostly focus on their health behaviors and demographic factors [17–19]. Few studies have been conducted on the use of medical services after survival among gastric cancer patients. Furthermore, studies examining medical service use according to the characteristics of rural and urban areas are still lacking. Such knowledge is essential to gain a deeper understanding of the readiness, accessibility, and quality of the current healthcare system [11].

Therefore, the Position Value for Relative Comparison (PARC) Index together with the National Health Insurance Service-National Sample Cohort (NHIS-NSC) data has been used in this study to compare the differences in medical service use between Korean gastric cancer patients before and after diagnosis while also analyzing regional differences for the same.

## Methods

### Data and study population

We used data from the NHIS-NSC database collected from 2002 to 2013 in Korea [20]. NHIS-NSC is an existing population-based cohort representing 2.2% of the total Korean population. In 2002, 1,025,340 people were randomly identified and followed for 11 years, with 1,014,730 remaining in 2013. The database contains personal details (e.g., age, gender, region of residence, income), medical treatment details (diagnosis codes from the 10th revision of the International Classification of Disease [ICD-10] and details of prescription), and general health examination data (i.e., laboratory test results, physical examinations, and answers to a questionnaire about lifestyle behaviors).

Our study inclusion criteria were cancer patients surviving 5 years or more after their diagnosis [21–23]. First, we extracted 9,598 cases of individuals diagnosed with gastric cancer (ICD-10 code: C16) between 2002 and 2013. Next, 2002–2004 was set as the washout period to ensure the exclusion of patients diagnosed before 2005. The 2002–2004 washout period was a stricter criterion because the NHIS database started in 2002 [24, 25]. In addition, to ensure a 5-year survival period after cancer diagnosis, participants who were first diagnosed with gastric cancer in 2010–2013 were excluded. Finally, our study included 1797 individuals who survived at least 5 years after diagnosis of gastric cancer.

All the data is provided in the NHIS-NSC database (https://nhiss.nhis.or.kr) and can be viewed upon reasonable request. The study protocol was approved by the Institutional Review Board of Yonsei University Severance Hospital (approval no: Y-2020–0031; Seoul, Republic of Korea). As this is a retrospective study, the requirement to obtain informed consent was waived.

### Variables

In this study, the independent variable of interest was healthcare-vulnerable region. The PARC index, which has been widely used in previous studies, was used to diagnose the level of medical care region-wise in Korea [26–28]. PARC is an objective indicator that can determine a relative position compared to other regions. The PARC value is between − 1 and 1, and when compared with the average value of the entire region, it is 1 for the best, 0 for the average, and − 1 for the worst [26]. This implies that if the PARC value is closer to − 1, the level of medical care in the area is lower than the average, whereas if the PARC value is closer to 1, the level of medical care in the area is higher than the average [26]. In this study, when the PARC value for a region was less than − 0.33, it was classified as a healthcare-vulnerable region [29].

The dependent variables of this study were the number of outpatient visits, hospitalization days, and number of emergency visits in a year, collectively referred to as medical service use. Possible confounding factors in this study were the variables that could affect medical service use among gastric cancer survivors [30]. These included sex, age, household income, medical insurance, diagnosis of disorder, Charlson comorbidity index, diagnosis of diabetes, and hypertension.

### Statistical analysis

Participants of this study comprised patients who had survived for at least 5 years after cancer diagnosis, and medical service use by each of them 2 years prior to the diagnosis was considered. Therefore, for each survivor who survived 5–7 years after cancer diagnosis, medical service use 2 years prior to diagnosis was set as the baseline and analyzed.

First, we performed a univariate analysis to analyze the difference in the number of outpatient visits, hospitalization days, and emergency use according to general characteristics, including healthcare-vulnerable regions, at the baseline of 5-year survivors after cancer diagnosis. Then, the medical service use among the patients who survived 5–7 years after cancer diagnosis and 2 years prior to the diagnosis was compared using *t* test and paired *t* test. In addition, the relationship with medical service use according to the healthcare-vulnerable region of the survivors 5 years after cancer diagnosis was analyzed using a generalized linear model. We analyzed the data using log link function and Poisson distribution based on the characteristics of the dependent variables [31]. Furthermore, an interaction term was used to consider the medical service use time in the relationship between healthcare vulnerability of a region and medical service use. All the data analyses were performed using SAS 9.4 software (SAS Institute Inc., Cary, NC, USA).

## Results

Among the patients diagnosed with gastric cancer, 1797 patients survived for at least 5 years post-diagnosis. Of these, 1541 patients (85.8%) belonged to healthcare-nonvulnerable regions, and 256 (14.2%) were from healthcare-vulnerable regions. For patients who survived at least 5 years post-diagnosis, the relationship with medical service use according to healthcare-vulnerable region was statistically significant for the number of outpatient and emergency visits. In addition, among the patients diagnosed with gastric cancer, 1368 patients survived for 6 years. Of these, 1156 (84.5%) and 212 (15.5%) patients belonged to healthcare-nonvulnerable regions and healthcare-vulnerable regions, respectively. For patients who survived for 6 years after cancer diagnosis, the relationship with medical service use according to healthcare-vulnerable region was statistically significant only for the number of outpatient visits. Finally, 928 patients survived for 7 years after diagnosis of gastric cancer. Of these, 792 patients (85.3%) belonged to healthcare-nonvulnerable regions, and 136 (14.7%) were from healthcare-vulnerable regions. For patients who survived for 7 years after cancer diagnosis, the relationship with medical service use according to healthcare-vulnerable region was statistically significant only for the number of outpatient visits (Supplementary Tables 1, 2, 3).

Table 1 shows the difference in medical service use over time among gastric cancer survivors between the healthcare-vulnerable and healthcare-nonvulnerable regions. Compared with the baseline, the number of outpatient visits in healthcare-vulnerable regions was 5.04 (standard deviation [SD], 27.3) times more after 5 years of gastric cancer survival and 5.37 (SD: 26.6) times more after 6 years, compared with the nonvulnerable regions; this difference was statistically significant.

**Table 1:** Differences in medical service use in terms of healthcare vulnerability

	Before^a^				After 5 years			Before^b^				After 6 years		
	*N*	%	Mean	SD	*N*	Mean	SD	*N*	%	Mean	SD	*N*	Mean	SD
Total	1797	100.0			1797			1368	100.0			1368		
Outpatient														
Nonvulnerable region	1541	85.8	15.30	19.0	1541	20.40	25.3	1156	64.3	14.17	16.3	1156	19.67	25.5
Vulnerable region	256	14.2	17.77	21.2	256	27.90	32.0	212	11.8	17.58	21.3	212	28.45	35.0
Differences of vulnerable region														
Inpatient														
Nonvulnerable region	1541	85.8	1.44	9.9	1541	6.35	33.0	1156	64.3	1.05	5.8	1156	7.10	37.4
Vulnerable region	256	14.2	1.87	10.9	256	7.58	29.1	212	11.8	1.59	10.0	212	8.79	34.0
Differences of vulnerable region														
ER														
Nonvulnerable region	1541	85.8	0.03	0.2	1541	0.08	0.5	1156	64.3	0.03	0.2	1156	0.10	0.6
Vulnerable region	256	14.2	0.05	0.2	256	0.16	0.6	212	11.8	0.05	0.2	212	0.17	0.7
Differences of vulnerable region														

	Before^c^				After 7 years			Unadjusted difference (before, after 5 years)			Unadjusted difference (before, after 6 years)			Unadjusted difference (before, after 7 years)		
	*N*	%	Mean	SD	*N*	Mean	SD	Estimate	SD	*P* value	Estimate	SD	*P* value	Estimate	SD	*P* value
Total	928	100.0			928											
Outpatient																
Nonvulnerable region	792	44.1	13.94	16.4	792	17.59	25.1	5.10	27.2	< .0001	5.50	25.6	< .0001	3.65	27.1	0.0002
Vulnerable region	136	7.6	16.57	23.2	136	24.37	38.6	10.14	27.8	< .0001	10.87	31.6	< .0001	7.79	33.5	0.0076
Differences of vulnerable region								5.04	27.3	0.0064	5.37	26.6	0.0201	4.15	28.2	0.1731
Inpatient																
Nonvulnerable region	792	44.1	1.17	6.2	792	6.30	33.0	4.91	34.5	< .0001	6.05	37.9	< .0001	5.14	33.7	< .0001
Vulnerable region	136	7.6	0.79	3.1	136	7.50	29.3	5.71	29.6	0.0023	7.20	35.2	0.0032	6.71	29.2	0.0082
Differences of vulnerable region								0.80	33.9	0.6974	1.15	37.5	0.6655	1.58	33.0	0.5704
ER	792	44.1	0.03	0.2	792	0.08	0.4	0.05	0.5	< .0001	0.07	0.6	0.0002	0.04	0.5	0.0077
Nonvulnerable region	136	7.6	0.03	0.2	136	0.15	0.6	0.12	0.6	0.0008	0.12	0.7	0.0120	0.13	0.6	0.0126
Vulnerable region								0.07	0.5	0.0782	0.05	0.6	0.2956	0.08	0.5	0.1230

^a^2 years before the onset of gastric cancer in survivors of 5 years after gastric cancer onset

^b^2 years before the onset of gastric cancer in survivors of 6 years after gastric cancer onset

^c^2 years before the onset of gastric cancer in survivors of 7 years after gastric cancer onset

*ER*, emergency room; *SD*, standard deviation

Table 2 shows the interaction effect between healthcare vulnerability and medical service use over time for 5-year gastric cancer survivors, after adjusting for all covariates. After 5 years of cancer survival compared to the baseline, there was a statistically significant difference in the number of outpatient visits (exp(β), 1.18; 95% CI [1.13–1.23]) in the healthcare-vulnerable region compared with the healthcare-nonvulnerable region.

**Table 2:** Medical service use interaction with healthcare vulnerability and year of medical service use

Variables	Outpatient			Inpatient			ER		
	exp(ß)	95% CI	*P* value	exp(ß)	95% CI	*P* value	exp(ß)	95% CI	*P* value
Healthcare-vulnerable area * year of medical use									
After 5 years^a^ × vulnerable region	1.18	(1.13–1.23)	< .0001	0.92	(0.82–1.02)	0.1224	1.26	(0.61–2.61)	0.5329
Before 2 years^b^ × nonvulnerable region	Ref			Ref			Ref		
Year of medical use									
After 5 years^a^	1.33	(1.31–1.36)	< .0001	4.42	(4.22–4.63)	< .0001	2.78	(1.98–3.91)	< .0001
Before 2 years^b^	Ref			Ref			Ref		
Healthcare vulnerability									
Nonvulnerable region	Ref			Ref			Ref		
Vulnerable region	1.06	(1.03–1.10)	0.0001	1.15	(1.04–1.27)	0.0059	1.51	(0.80–2.86)	0.2084
Sex									
Male	Ref			Ref			Ref		
Female	1.22	(1.20–1.23)	< .0001	1.35	(1.31–1.40)	< .0001	1.01	(0.76–1.33)	0.9558
Age									
< 50	Ref			Ref			Ref		
50–59	1.47	(1.43–1.50)	< .0001	2.23	(2.11–2.37)	< .0001	2.20	(1.42–3.42)	0.0005
≥ 60	1.81	(1.76–1.86)	< .0001	3.17	(2.98–3.39)	< .0001	3.05	(1.86–4.99)	< .0001
Household income									
Low	Ref			Ref			Ref		
Mid-low	0.84	(0.82–0.86)	< .0001	0.86	(0.81–0.91)	< .0001	0.91	(0.59–1.41)	0.6815
Mid-high	0.83	(0.81–0.85)	< .0001	0.83	(0.79–0.88)	< .0001	1.03	(0.67–1.57)	0.9001
Medical insurance									
NHI, self-employed	Ref			Ref			Ref		
NHI, employed	0.98	(0.96–0.99)	0.0028	0.99	(0.96–1.03)	0.684	0.86	(0.65–1.13)	0.2785
Medical aid	0.78	(0.73–0.84)	< .0001	3.55	(3.25–3.88)	< .0001	1.00	(0.35–2.91)	0.9957
Disorder^c^									
Yes	Ref			Ref			Ref		
No	0.83	(0.81–0.85)	< .0001	0.61	(0.58–0.64)	< .0001	0.76	(0.50–1.14)	0.1831
CCI									
0	Ref			Ref			Ref		
1	1.22	(1.19–1.25)	< .0001	0.72	(0.68–0.75)	< .0001	0.68	(0.48–0.98)	0.0386
2	1.48	(1.45–1.52)	< .0001	1.06	(1.01–1.11)	0.0179	0.70	(0.46–1.05)	0.0808
≥ 3	1.75	(1.71–1.80)	< .0001	1.14	(1.08–1.19)	< .0001	1.08	(0.75–1.57)	0.6716
Diabetes									
No	Ref			Ref			Ref		
Yes	1.32	(1.29–1.35)	< .0001	1.67	(1.61–1.74)	< .0001	1.86	(1.33–2.59)	0.0003
Hypertension									
No	Ref			Ref			Ref		
Yes	1.27	(1.25–1.29)	< .0001	1.69	(1.63–1.75)	< .0001	0.91	(0.68–1.23)	0.5473

Cancer survivors are those who survive 5 years after the onset of cancer

^a^Medical service use after 5 years of gastric cancer survival

^b^Medical service use 2 years before gastric cancer diagnosis

^c^Disorder refers to whether or not a disability is determined

*ER*, emergency room; *CI*, confidence interval; *NHI*, National Health Insurance; *CCI*, Charlson comorbidity index

Table 3 shows the results of analysis of medical service use according to the interaction between healthcare-vulnerable regions and the duration of 5–7 years after cancer survival compared with the baseline, after adjusting for all covariates. Compared with the baseline, there were statistically significant differences in the number of outpatient visits after 5 years (exp(β), 1.18; 95% CI [1.13–1.23]), 6 years (exp(β), 1.17; 95% CI [1.11–1.22]), and 7 years (exp(β), 1.17; 95% CI [1.10–1.24]) of cancer survival. In the case of hospitalization days, there was a statistically significant difference at 6 years (exp(β), 0.82; 95% CI [0.72–0.94]) and 7 years (exp(β), 1.76; 95% CI [1.43–2.18]) post-cancer survival. Furthermore, there was a tendency for the number of emergency visits to increase at 5 years (exp(β), 1.26; 95% CI [0.61–2.61]) and 7 years (exp(β), 2.19; 95% CI [0.68–7.02]) after cancer survival in healthcare-vulnerable regions; however, this result was not statistically significant (Table 3).

**Table 3:** Results of medical service use by the interaction of healthcare vulnerability and year of medical use (medical use at 5, 6, and 7 years after gastric cancer diagnosis)

Variables	Outpatient^*^			Inpatient^*^			ER^*^		
	exp(ß)	95% CI	*P* value	exp(ß)	95% CI	*P* value	exp(ß)	95% CI	*P* value
Healthcare vulnerability * year of medical use									
After 5 years × vulnerable region	1.18	(1.13–1.23)	< .0001	0.92	(0.82–1.02)	0.1224	1.26	(0.61–2.61)	0.5329
Before 2 years × nonvulnerable region	Ref			Ref			Ref		
Healthcare vulnerability * year of medical use									
After 6 years × vulnerable region	1.17	(1.11–1.22)	< .0001	0.82	(0.72–0.94)	0.0031	0.97	(0.44–2.11)	0.9383
Before 2 years × nonvulnerable region	Ref			Ref			Ref		
Healthcare vulnerability * year of medical use									
After 7 years × vulnerable region	1.17	(1.10–1.24)	< .0001	1.76	(1.43–2.18)	< .0001	2.19	(0.68–7.02)	0.1885
Before 2 years × nonvulnerable region	Ref			Ref			Ref		

^*^Adjusted for all covariate

*CI*, confidence interval; *ER*, emergency room

## Discussion

This study aimed to examine the existing regional disparities in the use of medical services available for gastric cancer patients who survived post-diagnosis, by considering medical service use before and after diagnosis. The results showed that there was a difference in medical service use region-wise among patients who survived at least 5 years after diagnosis. Compared with healthcare-nonvulnerable regions, in vulnerable regions, outpatient visits were more frequent from 5 years after cancer diagnosis, and hospitalization days decreased slightly in the sixth year of survival but increased rapidly in the seventh year. The number of emergency visits also showed a tendency to increase during 5–7 years after cancer diagnosis in healthcare-vulnerable regions compared with healthcare-nonvulnerable regions; however, this result was not statistically significant.

A complete agreement has not yet been reached on the relationship between medical service use and healthcare vulnerability of the region. According to previous overseas studies, the main conclusion was that patients in rural areas received less medical care owing to problems of accessibility than patients in cities, which was contrary to our results [32, 33]. Furthermore, a Korean study also found that cancer patients living in the metropolitan area had significantly higher medical service use, which was also contrary to our research results [34]. However, a study from Taiwan, which was somewhat similar to ours, found that cancer patients in rural areas were more likely to be admitted to hospice [35]. Furthermore, in a study examining the medical service use among cancer patients by categorizing Korea into metropolitan city, cities, and counties in the order of magnitude, it was found that a large city did not necessarily require significant medical service use [36]. The reason for such varied results in each of the preceding studies was because healthcare-vulnerable regions were defined differently in each of them. The differences in results may also be attributed to variations in country-specific policies or accessibilities.

The reason for more medical service use in Korea’s healthcare-vulnerable regions could be the high proportion of elderly population in these regions [37]. Although medical resources are concentrated in large cities, the high medical service use rate in healthcare-vulnerable regions can be interpreted in the following manner. In Korea, as the importance of cancer patient management has been highlighted, various cancer management programs have been introduced at the national level, and health insurance coverage has risen to approximately 80%. Therefore, medical options for treatment after diagnosis of cancer patients may have improved significantly compared with the past [38].

Moreover, in the case of Korea, our result may be attributed to the disparity in medical service use between regions being greatly alleviated compared with other countries, with access to big hospitals in large cities such as Seoul which has expanded due to the development of transportation such as Korean Train Express [38].

According to another previous study, it was confirmed that there was no significant difference in medical needs in Korea because the medical supply base for treating patients with severe diseases such as cancer was uniform across regions [36]. However, it was found that active treatment such as surgery for cancer patients was carried out in big medical institutions concentrated in large cities, whereas small- and medium-sized medical institutions in rural areas only provided passive hospitalization protection such as palliative/supportive non-invasive treatment [36].

There is no exact explanation for the sharp increase in hospitalization in the seventh year in this study; however, our results can be interpreted in several ways. Patients living in rural areas experienced delays in waiting time for receiving active treatment compared with those in metropolitan areas [39, 40]. Long waiting periods can adversely affect treatment outcomes, clearly leading to adverse effects on patient outcomes. In addition, it is well-known that the unequal distribution of medical institutions and personnel between urban and rural areas in Korea is a serious concern [13]. As a result, in the case of rural residents, the rate of cases not being treated in hospitals, despite suffering from diseases, is 25.9%, which is higher than 18.7% as reported for urban areas [13]. Furthermore, patients living in rural areas tend to visit local general practitioners; however, for specialist treatment in case of serious illness, they tend to travel to urban/metropolitan areas where general hospitals are concentrated [41]. As such, patients in rural areas do not receive timely treatment when a disease occurs; furthermore, owing to accessibility problems, mild disease may worsen and lead to hospitalization. Therefore, it is possible that the sudden increase in hospitalization may be attributed to these reasons [39].

Similarly, in our study, although not statistically significant, it was confirmed that the number of emergency visits increased in 5, 7 years after cancer diagnosis in healthcare-vulnerable regions. A possible explanation for this trend is that rural doctors spend less time in their clinics and clinics may close faster than urban areas [42]. Therefore, the use of emergency rooms (ERs) may increase in rural areas because there are significantly fewer alternatives to primary care. When these general clinics are not readily available, ERs can be an alternative to both minor and major urgent care [42]. Nevertheless, there have not been any detailed studies on the reasons for the high number of hospitalization and emergency visits for patients in healthcare-vulnerable regions. Therefore, future studies should examine these reasons in more detail.

Overall, it was confirmed that the medical service use among patients living in healthcare-vulnerable and nonvulnerable regions at least 5 years after gastric cancer diagnosis may differ depending on whether they reside in healthcare-vulnerable regions. However, in our study, the detailed reasons for medical service use could not be considered. Therefore, future studies should clarify the reasons for medical care use, aiming at revealing the factors related to disparity in medical service use and the difference in health outcomes.

### Limitations

Our study has several limitations. First, information on cancer stage or severity could not be incorporated into the analysis due to data limitations. To overcome these obstacles and enhance the homogeneity of the study population, only patients who survived at least 5 years after diagnosis of gastric cancer were included. However, to accurately understand the medical service use among cancer survivors, further research is needed to determine the relationship between medical service use among cancer survivors and whether they reside in a healthcare-vulnerable region, considering the severity at the time of diagnosis. Second, because this study used insurance claims data, it was not possible to incorporate several potential covariates, including education level, health literacy rate, and household size. Therefore, the potential presence of residual confounding factors could not be completely excluded. However, relevant demographic and health-related factors were incorporated, including disability status and comorbidities. Third, the use of administrative claims data relates to specific issues. For example, reliance on ICD-10 codes to determine comorbidity can lead to misclassification due to incorrect coding behavior. Furthermore, the inaccuracy of the ICD-10 codes for diagnosis may have produced some misclassifications, including incorrect coding of the data by the original coders due to the nature of the insurance claims data. Fourth, in this study, detailed information on medical service use could not be identified. Our goal was to provide basic data on medical service use among cancer survivors depending on whether they were healthcare-vulnerable region. Therefore, in future studies, it is necessary to consider not only detailed forms of medical service use and places of medical use, but also other types of medical services such as psychosocial support.

Despite these limitations, there are several advantages to our research. First, this study used sample data representing Koreans across all regions of Korea [20]. Although our dataset is only 2% of the total population and the results are not generalizable to other countries, this study highlights the difference in medical service use among gastric cancer survivors between the healthcare-vulnerable and nonvulnerable regions. Second, we did not simply divide the healthcare-vulnerable region into urban and rural areas but classified them based on PARC scores. Therefore, our classification may have been more accurate compared with previous studies. Third, to the best of our knowledge, this study is the first to investigate the relationship between healthcare vulnerability of a region and medical service use by additionally considering the concept of time before and after diagnosis of gastric cancer. Consequently, our results are more sophisticated than previous studies.

## Conclusions

Our findings suggest that gastric cancer survivors living in healthcare-vulnerable regions are more likely to have increased medical service use 5 years after diagnosis compared with patients living in healthcare-nonvulnerable regions. These results also suggest a significant increase in healthcare disparities over time. Therefore, in future research, additional research is needed to elucidate the cause of the disparity in healthcare use and the result of the difference in health outcomes.

## Supplementary Information

Below is the link to the electronic supplementary material.

Supplementary file1 (DOCX 54 KB)

## Data Availability

All the data used in this study are provided in the National Health Insurance Corporation database (https://nhiss.nhis.or.kr) and can be viewed upon reasonable request.
