# Supplementary material for: Difference in medical service use among Korean gastric cancer survivors according to regional healthcare vulnerabilities: a cohort study
Source: Support Care Cancer. 2022 Sep 3;30(11):9233–41. doi: 10.1007/s00520-022-07346-2 (PMC9633440; doi:10.1007/s00520-022-07346-2)
Supplement: Supplementary file 1 — Supplementary file1 (DOCX 54 KB) [file 520_2022_7346_MOESM1_ESM.docx]

***Supportive Care in Cancer***

**Difference in Medical Service Use among Korean Gastric Cancer Survivors according to Regional Healthcare Vulnerabilities: A Cohort Study**

Sung Hoon Jeong ^1,2^, Jae Hong Joo ^1,2^, Minah Park ^1,2^, Choa Yun ^4^, Soo Hyun Kang ^1,2^, Eun-Cheol Park ^2,3^, *Yoon Dae Han ^5^, *Sung-In Jang

***Corresponding Author**:

Sung In Jang, MD, PhD

Department of Preventive Medicine and Institute of Health Services Research, Yonsei University College of Medicine, 50 Yonsei-ro, Seodaemun-gu, Seoul 03722, Korea.

Tel.: +82-2-2228-1863, Fax: +82-2-392-8133

Email: [jangsi@yuhs.ac](mailto:jangsi@yuhs.ac)

***Corresponding Author:**

Yoon Dae Han, MD, PhD

Division of Colorectal Surgery, Department of Surgery, Severance Hospital, Yonsei University College of Medicine,

50-1 Yonsei-ro Seodaemun-gu, Seoul, 03722, Republic of Korea

Tel: +82-2-2228-2128; E-mail: ACYLYOON@yuhs.ac; Fax: +82-2-313-8289

| **Supplementary Table 1.** General characteristics of patients who survived at least five years post cancer diagnosis | | | | | | | | | | | | | | |
| --- | --- | --- | --- | --- | --- | --- | --- | --- | --- | --- | --- | --- | --- | --- |
|  | **Outpatient** | | | | |  | **Inpatient** | | |  | **ER** | | | |
|  | **N** | **%** | **Mean** | **SD** | ***P*-value** |  | **Mean** | **SD** | ***P*-value** |  | **Mean** | **SD** | ***P*-value** |  |
| **Total** | 1,797 | 100.0 |  |  |  |  |  |  |  |  |  |  |  |  |
| **Healthcare Vulnerability** | | |  |  | <.0001 |  |  |  | 0.5717 |  |  |  | 0.0107 |  |
| Non-vulnerable region | 1,541 | 85.8 | 20.4 | 25.3 |  |  | 6.3 | 33.0 |  |  | 0.08 | 0.5 |  |  |
| Vulnerable region | 256 | 14.2 | 27.9 | 32.0 |  |  | 7.6 | 29.1 |  |  | 0.16 | 0.6 |  |  |
| **Sex** |  |  |  |  | 0.2529 |  |  |  | 0.3674 |  |  |  | 0.6585 |  |
| Male | 1,141 | 63.5 | 20.9 | 27.4 |  |  | 6.0 | 28.6 |  |  | 0.10 | 0.5 |  |  |
| Female | 656 | 36.5 | 22.4 | 24.9 |  |  | 7.4 | 38.4 |  |  | 0.09 | 0.4 |  |  |
| **Age** |  |  |  |  | <.0001 |  |  |  | 0.0015 |  |  |  | 0.0250 |  |
| <50 | 457 | 25.4 | 13.1 | 14.6 |  |  | 2.4 | 15.8 |  |  | 0.04 | 0.3 |  |  |
| 50-59 | 1,027 | 57.2 | 23.1 | 26.9 |  |  | 7.1 | 34.2 |  |  | 0.10 | 0.5 |  |  |
| ≥60 | 313 | 17.4 | 28.4 | 34.7 |  |  | 10.6 | 42.8 |  |  | 0.14 | 0.5 |  |  |
| **Household income** | | |  |  | 0.1013 |  |  |  | 0.5075 |  |  |  | 0.7559 |  |
| Low | 229 | 12.7 | 25.3 | 37.3 |  |  | 9.0 | 32.1 |  |  | 0.12 | 0.6 |  |  |
| Mid-low | 724 | 40.3 | 21.1 | 26.2 |  |  | 6.2 | 35.8 |  |  | 0.09 | 0.5 |  |  |
| Mid-high | 844 | 47.0 | 20.8 | 23.0 |  |  | 6.1 | 29.5 |  |  | 0.09 | 0.4 |  |  |
| **Medical Insurance** | | |  |  | 0.8297 |  |  |  | 0.0103 |  |  |  | 0.8271 |  |
| NHI, self-employed | 692 | 38.5 | 21.3 | 26.9 |  |  | 5.6 | 27.2 |  |  | 0.10 | 0.5 |  |  |
| NHI, employed | 1,073 | 59.7 | 21.6 | 26.1 |  |  | 6.6 | 34.1 |  |  | 0.09 | 0.5 |  |  |
| Medical Aid | 32 | 1.8 | 21.5 | 30.9 |  |  | 23.2 | 65.1 |  |  | 0.13 | 0.5 |  |  |
| **Disorder^a^** |  |  |  |  | 0.2122 |  |  |  | 0.3547 |  |  |  | 0.8695 |  |
| No | 1,645 | 91.5 | 21.1 | 26.5 |  |  | 6.2 | 31.1 |  |  | 0.09 | 0.5 |  |  |
| Yes | 152 | 8.5 | 25.3 | 26.7 |  |  | 9.8 | 44.9 |  |  | 0.11 | 0.4 |  |  |
| **CCI** |  |  |  |  | <.0001 |  |  |  | 0.3803 |  |  |  | 0.7077 |  |
| 0 | 459 | 25.5 | 16.1 | 21.0 |  |  | 5.8 | 33.4 |  |  | 0.10 | 0.7 |  |  |
| 1 | 628 | 34.9 | 18.9 | 21.6 |  |  | 4.7 | 25.0 |  |  | 0.07 | 0.4 |  |  |
| 2 | 375 | 20.9 | 24.6 | 27.7 |  |  | 8.1 | 37.8 |  |  | 0.09 | 0.4 |  |  |
| ≥3 | 335 | 18.6 | 30.1 | 36.1 |  |  | 9.2 | 36.9 |  |  | 0.12 | 0.4 |  |  |
| **Diabetes** |  |  |  |  | 0.0148 |  |  |  | 0.0448 |  |  |  | 0.0773 |  |
| Yes | 206 | 11.5 | 28.4 | 30.2 |  |  | 11.9 | 46.0 |  |  | 0.16 | 0.6 |  |  |
| No | 1,591 | 88.5 | 20.6 | 25.9 |  |  | 5.8 | 30.3 |  |  | 0.08 | 0.5 |  |  |
| **Hypertension** |  |  |  |  | 0.0108 |  |  |  | 0.007 |  |  |  | 0.3631 |  |
| Yes | 536 | 29.8 | 27.2 | 31.6 |  |  | 11.1 | 49.4 |  |  | 0.10 | 0.5 |  |  |
| No | 1,261 | 70.2 | 19.0 | 23.6 |  |  | 4.6 | 21.4 |  |  | 0.09 | 0.5 |  |  |
| ^a^Disorder refers to whether or not a disability is determined. | | | | | | | | | | | | | | |
| ER, Emergency room; SD Standard Deviation; NHI National health insurance; CCI Charlson comorbidity index | | | | | | | | | | | | | | |

| **Supplementary Table 2.** General characteristics of patients who survived six years post cancer diagnosis | | | | | | | | | | | | | | | | | | | | | | | | | | |
| --- | --- | --- | --- | --- | --- | --- | --- | --- | --- | --- | --- | --- | --- | --- | --- | --- | --- | --- | --- | --- | --- | --- | --- | --- | --- | --- |
|  | **Outpatient** | | | | | | | | | |  | | **Inpatient** | | | | | |  | | **ER** | | | | | |
|  | **N** | | **%** | | **Mean** | | **SD** | | ***P*-value** | |  | | **Mean** | | **SD** | | ***P*-value** | |  | | **Mean** | | **SD** | | ***P*-value** | |
| **Total** | 1,368 | | 100.0 | |  | |  | |  | |  | |  | |  | |  | |  | |  | |  | |  | |
| **Healthcare Vulnerability** | | | | | | |  | | <.0001 | |  | |  | |  | | 0.5390 | |  | |  | |  | | 0.0867 | |
| Non-vulnerable region | 1,156 | | 84.5 | | 19.67 | | 25.5 | |  | |  | | 7.10 | | 37.4 | |  | |  | | 0.10 | | 0.6 | |  | |
| Vulnerable region | 212 | | 15.5 | | 28.45 | | 35.0 | |  | |  | | 8.79 | | 34.0 | |  | |  | | 0.17 | | 0.7 | |  | |
| **Sex** |  | |  | |  | |  | | 0.4118 | |  | |  | |  | | 0.7276 | |  | |  | |  | | 0.1576 | |
| Male | 857 | | 62.6 | | 21.46 | | 29.8 | |  | |  | | 7.09 | | 34.8 | |  | |  | | 0.13 | | 0.7 | |  | |
| Female | 511 | | 37.4 | | 20.33 | | 22.7 | |  | |  | | 7.82 | | 40.2 | |  | |  | | 0.08 | | 0.4 | |  | |
| **Age** |  | |  | |  | |  | | <.0001 | |  | |  | |  | | 0.0151 | |  | |  | |  | | 0.0605 | |
| <50 | 366 | | 26.8 | | 12.35 | | 13.8 | |  | |  | | 3.57 | | 26.2 | |  | |  | | 0.06 | | 0.6 | |  | |
| 50‒59 | 788 | | 57.6 | | 23.55 | | 28.5 | |  | |  | | 7.65 | | 37.8 | |  | |  | | 0.11 | | 0.6 | |  | |
| ≥60 | 214 | | 15.6 | | 26.64 | | 36.1 | |  | |  | | 12.79 | | 47.2 | |  | |  | | 0.19 | | 0.7 | |  | |
| **Household income** | | | | | | |  | | 0.0126 | |  | |  | |  | | 0.4338 | |  | |  | |  | | 0.1228 | |
| Low | 181 | | 13.2 | | 24.14 | | 32.8 | |  | |  | | 10.51 | | 42.7 | |  | |  | | 0.19 | | 1.0 | |  | |
| Mid-low | 532 | | 38.9 | | 22.88 | | 29.4 | |  | |  | | 7.40 | | 36.6 | |  | |  | | 0.11 | | 0.6 | |  | |
| Mid-high | 655 | | 47.9 | | 18.68 | | 23.6 | |  | |  | | 6.46 | | 35.4 | |  | |  | | 0.09 | | 0.4 | |  | |
| **Medical Insurance** | | | | | | |  | | 0.5664 | |  | |  | |  | | 0.1469 | |  | |  | |  | | 0.1882 | |
| NHI, self-employed | 527 | | 38.5 | | 21.08 | | 27.8 | |  | |  | | 8.43 | | 42.0 | |  | |  | | 0.13 | | 0.7 | |  | |
| NHI, employed | 816 | | 59.6 | | 21.08 | | 27.3 | |  | |  | | 6.32 | | 32.7 | |  | |  | | 0.09 | | 0.5 | |  | |
| Medical Aid | 25 | | 1.8 | | 18.56 | | 22.0 | |  | |  | | 19.00 | | 47.7 | |  | |  | | 0.28 | | 0.8 | |  | |
| **Disorder^a^** | | | | |  | |  | | 0.0853 | |  | |  | |  | | 0.5396 | |  | |  | |  | | 0.1511 | |
| No | 1,249 | | 91.3 | | 20.49 | | 27.1 | |  | |  | | 7.08 | | 36.6 | |  | |  | | 0.10 | | 0.6 | |  | |
| Yes | 119 | | 8.7 | | 26.80 | | 29.6 | |  | |  | | 10.35 | | 39.7 | |  | |  | | 0.21 | | 1.0 | |  | |
| **CCI** |  | |  | |  | |  | | <.0001 | |  | |  | |  | | 0.0708 | |  | |  | |  | | 0.7436 | |
| 0 | 370 | | 27.0 | | 15.72 | | 21.7 | |  | |  | | 7.61 | | 40.2 | |  | |  | | 0.11 | | 0.7 | |  | |
| 1 | 488 | | 35.7 | | 18.54 | | 23.1 | |  | |  | | 4.80 | | 23.2 | |  | |  | | 0.08 | | 0.5 | |  | |
| 2 | 277 | | 20.2 | | 24.18 | | 28.8 | |  | |  | | 6.49 | | 34.6 | |  | |  | | 0.12 | | 0.7 | |  | |
| ≥3 | 233 | | 17.0 | | 30.96 | | 37.4 | |  | |  | | 13.37 | | 53.4 | |  | |  | | 0.15 | | 0.6 | |  | |
| **Diabetes** |  | |  | |  | |  | | 0.0739 | |  | |  | |  | | 0.1137 | |  | |  | |  | | 0.7955 | |
| Yes | 141 | | 10.3 | | 27.94 | | 34.4 | |  | |  | | 13.45 | | 58.1 | |  | |  | | 0.13 | | 0.6 | |  | |
| No | 1,227 | | 89.7 | | 20.24 | | 26.3 | |  | |  | | 6.66 | | 33.6 | |  | |  | | 0.11 | | 0.6 | |  | |
| **Hypertension** |  | |  | |  | |  | | 0.073 | |  | |  | |  | | 0.1048 | |  | |  | |  | | 0.0321 | |
| Yes | 383 | | 28.0 | | 26.76 | | 31.7 | |  | |  | | 11.69 | | 51.4 | |  | |  | | 0.07 | | 0.4 | |  | |
| No | 985 | | 72.0 | | 18.81 | | 25.2 | |  | |  | | 5.68 | | 29.2 | |  | |  | | 0.12 | | 0.7 | |  | |
| ^a^Disorder refers to whether or not a disability is determined. | | | | | | | | | | | | | | | | | | | | | | | | | | |
| ER, Emergency room; SD Standard Deviation; NHI National health insurance; CCI Charlson comorbidity index | | | | | | | | | | | | | | | | | | | | | | | | | | |
| **Supplementary Table 3.** General characteristics of patients who survived seven years post cancer diagnosis | | | | | | | | | | | | | | | | | | | | | | | | | |  |
|  | | **Outpatient** | | | | | | | |  | | **Inpatient** | | | | | |  | | **ER** | | | | | |  |
|  |  | **N** | | **%** | | **Mean** | **SD** | ***P*-value** | |  | | **Mean** | | **SD** | | ***P*-value** | |  | | **Mean** | | **SD** | | ***P*-value** | |  |
| **Total** | | 928 | | 100.0 | |  |  |  | |  | |  | |  | |  | |  | |  | |  | |  | |  |
| **Healthcare Vulnerability** | | | | | | |  | 0.0072 | |  | |  | |  | | 0.6872 | |  | |  | |  | | 0.0607 | |  |
| Non-vulnerable region | | 792 | | 85.3 | | 17.59 | 25.1 |  | |  | | 6.30 | | 33.0 | |  | |  | | 0.08 | | 0.4 | |  | |  |
| Vulnerable region | | 136 | | 14.7 | | 24.37 | 38.6 |  | |  | | 7.50 | | 29.3 | |  | |  | | 0.15 | | 0.6 | |  | |  |
| **Sex** | |  | |  | |  |  | 0.6006 | |  | |  | |  | | 0.0688 | |  | |  | |  | | 0.7111 | |  |
| Male | | 582 | | 62.7 | | 18.89 | 29.5 |  | |  | | 4.99 | | 26.4 | |  | |  | | 0.08 | | 0.5 | |  | |  |
| Female | | 346 | | 37.3 | | 18.06 | 24.2 |  | |  | | 8.98 | | 40.5 | |  | |  | | 0.10 | | 0.4 | |  | |  |
| **Age** | |  | |  | |  |  | <.0001 | |  | |  | |  | | <.0001 | |  | |  | |  | | 0.0002 | |  |
| <50 | | 262 | | 28.2 | | 11.23 | 15.5 |  | |  | | 0.80 | | 2.8 | |  | |  | | 0.02 | | 0.1 | |  | |  |
| 50-59 | | 530 | | 57.1 | | 21.34 | 29.3 |  | |  | | 6.77 | | 30.4 | |  | |  | | 0.09 | | 0.4 | |  | |  |
| ≥60 | | 136 | | 14.7 | | 21.97 | 35.5 |  | |  | | 16.29 | | 58.6 | |  | |  | | 0.22 | | 0.8 | |  | |  |
| **Household income** | | | | | | |  | 0.1475 | |  | |  | |  | | 0.9709 | |  | |  | |  | | 0.4467 | |  |
| Low | | 123 | | 13.3 | | 18.22 | 31.1 |  | |  | | 7.46 | | 39.9 | |  | |  | | 0.08 | | 0.4 | |  | |  |
| Mid-low | | 373 | | 40.2 | | 20.73 | 31.0 |  | |  | | 6.35 | | 27.2 | |  | |  | | 0.11 | | 0.5 | |  | |  |
| Mid-high | | 432 | | 46.6 | | 16.83 | 23.0 |  | |  | | 6.31 | | 34.2 | |  | |  | | 0.07 | | 0.4 | |  | |  |
| **Medical Insurance** | | | | | | |  | 0.393 | |  | |  | |  | | 0.9885 | |  | |  | |  | | 0.9558 | |  |
| NHI, self-employed | | 363 | | 39.1 | | 19.88 | 32.7 |  | |  | | 5.84 | | 27.6 | |  | |  | | 0.08 | | 0.4 | |  | |  |
| NHI, employed | | 546 | | 58.8 | | 17.81 | 24.0 |  | |  | | 6.97 | | 35.7 | |  | |  | | 0.10 | | 0.5 | |  | |  |
| Medical Aid | | 19 | | 2.0 | | 15.89 | 17.5 |  | |  | | 4.58 | | 14.9 | |  | |  | | 0.05 | | 0.2 | |  | |  |
| **Disorder^a^** | |  | |  | |  |  | 0.2341 | |  | |  | |  | | 0.4689 | |  | |  | |  | | 0.0571 | |  |
| No | | 847 | | 91.3 | | 18.15 | 26.9 |  | |  | | 6.23 | | 31.4 | |  | |  | | 0.08 | | 0.4 | |  | |  |
| Yes | | 81 | | 8.7 | | 23.12 | 33.5 |  | |  | | 9.11 | | 41.8 | |  | |  | | 0.19 | | 0.7 | |  | |  |
| **CCI** | |  | |  | |  |  | 0.059 | |  | |  | |  | | 0.0207 | |  | |  | |  | | 0.4709 | |  |
| 0 | | 270 | | 29.1 | | 14.50 | 22.9 |  | |  | | 4.53 | | 23.7 | |  | |  | | 0.05 | | 0.3 | |  | |  |
| 1 | | 339 | | 36.5 | | 18.48 | 28.0 |  | |  | | 4.17 | | 25.2 | |  | |  | | 0.09 | | 0.5 | |  | |  |
| 2 | | 180 | | 19.4 | | 20.36 | 30.2 |  | |  | | 6.67 | | 39.4 | |  | |  | | 0.08 | | 0.3 | |  | |  |
| ≥3 | | 139 | | 15.0 | | 24.44 | 30.4 |  | |  | | 15.65 | | 47.9 | |  | |  | | 0.15 | | 0.6 | |  | |  |
| **Diabetes** | |  | |  | |  |  | 0.3853 | |  | |  | |  | | 0.418 | |  | |  | |  | | 0.5225 | |  |
| Yes | | 85 | | 9.2 | | 23.09 | 32.9 |  | |  | | 10.93 | | 43.0 | |  | |  | | 0.13 | | 0.7 | |  | |  |
| No | | 843 | | 90.8 | | 18.13 | 27.0 |  | |  | | 6.03 | | 31.2 | |  | |  | | 0.08 | | 0.4 | |  | |  |
| **Hypertension** | |  | |  | |  |  | 0.2804 | |  | |  | |  | | 0.3946 | |  | |  | |  | | 0.384 | |  |
| Yes | | 238 | | 25.6 | | 22.89 | 30.3 |  | |  | | 10.72 | | 43.8 | |  | |  | | 0.14 | | 0.5 | |  | |  |
| No | | 690 | | 74.4 | | 17.09 | 26.5 |  | |  | | 5.01 | | 27.3 | |  | |  | | 0.07 | | 0.4 | |  | |  |
| ^a^Disorder refers to whether or not a disability is determined. | | | | | | | | | | | | | | | | | | | | | | | |  | |  |
| ER, Emergency room; SD Standard Deviation; NHI National health insurance; CCI Charlson comorbidity index | | | | | | | | | | | | | | | | | | | | | | | |  | |  |
